# Influence of Magnetic Water on Concrete Properties with Different Magnetic Field Exposure Times

**DOI:** 10.3390/ma15124291

**Published:** 2022-06-17

**Authors:** Malathy Ramalingam, Karuppasamy Narayanan, Arivoli Masilamani, Parthiban Kathirvel, Gunasekaran Murali, Nikolai Ivanovich Vatin

**Affiliations:** 1 Department of Civil Engineering, Sona College of Technology, Salem 636005, Tamil Nadu, India; malathycivil@sonatech.ac.in (M.R.); arivolisenthil@gmail.com (A.M.); 2 School of Civil Engineering, SASTRA Deemed University, Thirumalaisamudram, Thanjavur 613401, Tamil Nadu, India; 3 Peter the Great St. Petersburg Polytechnic University, 195251 St. Petersburg, Russia; murali_22984@yahoo.com (G.M.); vatin@mail.ru (N.I.V.)

**Keywords:** magnetic water, workability, compressive strength, magnetic field, X-ray diffraction

## Abstract

The characteristics of a concrete mix are purely dependent on the hydration of cement that is carried forward by using the water quality used in the mix. Several researchers have focused on incorporating pozzolanic or nanomaterials to improve the hydration mechanisms and impart high strength to concrete. A new technology has been introduced to improve the properties of concrete by magnetic-field-treated water (MFTW). Due to magnetization, water particles become charged and the molecules inside the water cluster decrease from 13 to 5 or 6, which eventually decreases the hardness of water, thus improving the strength of concrete when compared to the use of normal water (NW). In advanced construction techniques and practices, the application of Magnetic Water (MW) plays an important role in boosting physicochemical properties. This research work focused on evaluating the standards of water quality through physiochemical analysis, such as Electrical Conductivity (EC), Viscosity, pH, and Total Dissolved Solids (TDS) with the MW at different exposure periods (60 min (MW60), 45 min (MW45), 30 min (MW30), 15 min (MW15), and instant exposure (MWI)). Experiments were carried out to evaluate the fresh, hardened, and microstructural behavior of concrete made with magnetic water (MW) using a permanent magnet of PERMAG (N407) under a field intensity of 0.9 Tesla. In addition, optical properties such as X-ray Diffraction (XRD) and Ultraviolet (UV) absorption were considered for the MW60 mix to ensure water magnetization. Characterization methods such as Fourier Transform Infrared Spectroscopy (FT-IR), Thermogravimetric Analysis (TGA), and Scanning Electron Microscopy (SEM) were employed for NWC and MWC to quantify the hydrated products. From the results, it was observed that the magnetic effect on water characteristics showed significant improvement in the concrete properties with the increase in exposure duration. There were increments of 25.6% and 24.1% in workability and compressive strength, respectively, for the MW60 mix compared to normal water concrete (NWC).

## 1. Introduction

Concrete is a standard construction material, and during production, it consumes tons of freshwater each year [1]. Several works have been carried out to evaluate the improved performance of concrete under the influence of incorporating carbon nanotubes [2,3,4], and subsequent models have been developed to estimate the variables influencing the properties of concrete. Because of their favorable properties (e.g., compressive strength, stiffness, and durability), commonly accessible ingredients, and low cost, cementitious materials are widely employed in buildings, and cement is a binding material in concrete, which needs water to react. Thus, the quality and quantity of water used in concrete must be carefully decided for making high-quality concrete [5]. More than 70% of the Earth’s crust contains water, in which 97% of Earth’s water is in seas and oceans, with the remaining 3% as fresh water. In this freshwater, around 75% is available as glaciers, 24.5% as groundwater, 0.04% as atmospheric water vapor, and nearly 0.34% as rivers that are used for electricity generation [6] and drinking purposes. If this water used in the construction industry continues, there will be a scarcity in drinking water and water free from salts [7]. Developing countries such as India are still suffering due to the unavailability of fresh water and contamination of water that indirectly affects millions of people [8]. Due to this, obtaining potable water for mixing concrete is a big concern. As a replacement to the potable water, groundwater, bore well water, and treated seawater are being used for construction purposes [9]. However, the water collected from these sources may be highly acidic, and base concentrations that are vibrant in reacting with the cement composition will directly impact the strength of concrete. IS 456-2000 [10] specifies the presence of organic salts, suspended salts, acids, alkalis, algae, and oil contamination in water within the allowable limit, which can be used for mixing concrete [11]. The construction industry relies on nearby available well water or tap water. Enforcing the magnetic field, the water hardness potential affects the colloids by internal separation. This greatly impacts the magnetic intensity and duration of exposure of water to magnetic fields [12]. There is also another solution to extract wastewater from dyeing, the textile industry, and tanneries that have to be recycled to make it suitable for concrete making. Hence, this has emerged as a new technology to produce clean water [13]. The Magnetic-Field-Treated Water (MFTW) technology is one solution to overcome the scarcity of potable water and excessive usage of locally available water. Here, water becomes magnetized after passing through the magnetic field. Due to magnetization, the optical properties of water and infrared absorption property become altered [14]. In addition, the physiochemical properties of the MW are considerably changed due to magnetization. For instance, water conductivity is increased, but the surface tension is decreased [15,16]. In addition, the frictional coefficient of the MW is lower than that of the normal water (NW) [17]. In [18,19,20], the mechanism of magnetization and its effect on the hydrogen bonds of water were presented. The magnetic field reduces the effect of chemical additives on the cement composition. In other words, due to magnetization, large water clusters are broken down to form smaller clusters or single water molecules. It also decreases the bond angle from 104.5° to 103° [21,22,23]. In addition, water treated with magnetic fields is used in various public sectors for direct use with the blending nature of the magnetic field in the water, increasing the evaporation rate [24]. MFTW plays a vital role in controlling and ameliorating dispersion separation [25,26,27]. The concrete’s physiochemical and mechanical properties depend on the quality of raw materials and the quality and quantity of water. MW enhances the hydration process of cement by repelling the cement particles [28], with no clear idea of how to enhance the property of the MW and no strong report or test methods to confirm whether the water is magnetized.

For the past few decades, the physiochemical properties of water have changed by using magnetic action [29]. Many researchers have conducted studies on the magnetic field and conflicting results have been stated based on the scientific outcome in the water [30,31,32,33]. Some studies have reported that the property of water with respect to surface tension is decreased when there is an increment in viscosity [18,34]. The changing effect of the magnetic field on the molecular and atomic structures of water has been investigated by many researchers. From the infrared spectrum techniques, Raman, UV-visible, and X-ray radiation showed that the efficiency of the magnetic field depends on magnetizing time and temperature of the water. Temperature plays a predominant role in fluctuating the measurement of molecules and atoms in the water [35].

Numerous findings have reported that there is only a change in physiochemical properties of water due to magnetization. However, the properties related to the suitability of magnetized water used to mix concrete with respect to fresh and hardened properties is scarce in the literature and hence needs to be explored. Hence, this paper focuses on the physiochemical properties of water such as pH, Total Dissolved Solids (TDS), Electrical Conductivity (EC), and viscosity before and after magnetization with different exposure times, and the results are compared. Here, locally available NW was considered, and the magnetized water was prepared using the permanent magnet of 0.9 Tesla intensity at different exposure periods (60 min (MW60), 45 min (MW45), 30 min (MW30), 15 min (MW15), and instant exposure (MWI)), the role of the applied magnetic field was maintained from instant magnetic water exposure (MWI) to magnetic exposure times up to 60 min (MW60) at an interval of 15 min, and the results were optimized. At the optimized time of magnetic field exposure, the MW was tested using X-ray Diffraction (XRD) and Ultraviolet (UV) absorption to confirm the magnetization of water. Then, the MW was used to prepare M20-grade concrete, and its effect on fresh and hardened properties was evaluated. To validate the enhanced hydration process, thermogravimetric analysis (TGA), Scanning Electron Microscopy (SEM), and Fourier Transform Infrared Spectrophotometer (FT-IR) studies were conducted on concrete powder samples. The significant contributions of this paper are to replace normal tap water with magnetically treated water in the preparation of concrete mixes and to evaluate its effect on fresh and hardened concrete properties through water magnetization.

## 2. Experimental Program

### 2.1. Magnetic Water Preparation

**Figure 1:**
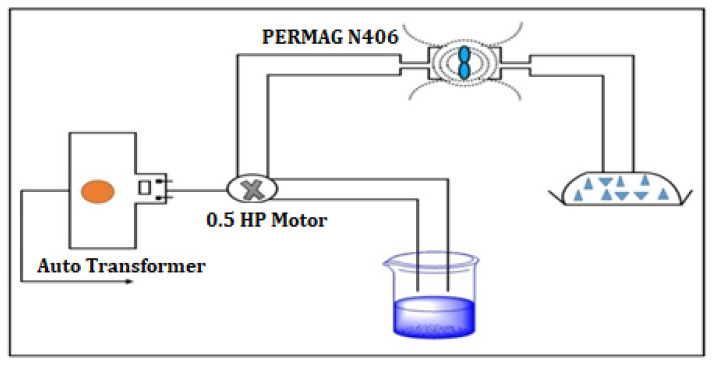
Schematic diagram of MW setup.

To prepare the magnetic water (MW), the NW was passed through the magnet PERMAG Neodymium (N406) with 0.9 Tesla intensity, as well as with a 0.075 m/s flow velocity, for different exposure periods (60 min (MW60), 45 min (MW45), 30 min (MW30), 15 min (MW15), and instant exposure (MWI)). Figure 1 shows a schematic diagram of the experimental setup, which consists of a 0.5 HP motor that works on shifting the available water in a container and makes it pass through the magnetic flux attached to the tube. The PERMAG plays a significant role in developing an intense and well-focused magnetic field for the water flowing through the pipeline.

### 2.2. Methodology

**Figure 2:**
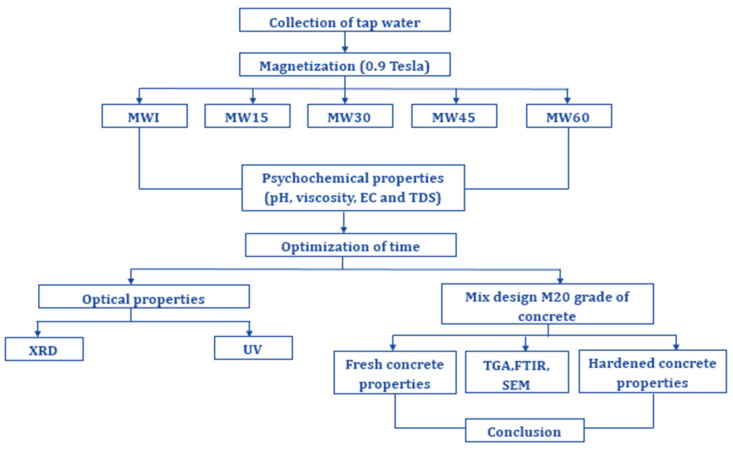
Research framework.

The NW used in this experimental study was collected from local sources for mixing concrete. Previous studies have shown that the magnetic exposure time tends to increase the workability of the concrete mix due to the influence of the magnetic field on cluster molecules of water. As not much strength gain was found with respect to the increase in contact or exposure time, in view of the time and energy consumption for the preparation of magnetized water, this work was restricted to evaluate the performance of magnetized water on concrete element up to 60 min with 15 min intervals. These samples were magnetized with a PERMAG N406 with 0.9 Tesla field intensity with respect to the exposure times of 60 min (MW60), 45 min (MW45), 30 min (MW30), 15 min (MW15), and instant exposure (MWI). The physiochemical parameters, namely pH, TDS, viscosity, and EC, were obtained before and after magnetization for different exposure periods. Optical analyses such as XRD and UV were performed with NW and MW from the optimized exposure period. Further, to incorporate the MW with concrete, a mix proportion was developed for M20 grade with a mix ratio of 1:1.82:3.10 in accordance to IS 10262:2009 [36], and the corresponding magnetization effect in terms of workability and strength was studied. In addition, the development of hydrated products with NW and MW was examined through TGA and FTIR studies and the effect of crystal growth on concrete was also analyzed using SEM analysis. Figure 2 shows the detailed methodology of the developed framework.

### 2.3. Physiochemical Properties

In a water molecule, two hydrogen atoms are linked by using a single chemical bond to an oxygen atom at an angle of 104.5°. The atomic structure of hydrogen molecules in water has $H^{+}$ and ${\mathrm{OH}}^{-}$ ions. Here, the magnetic effect directly relates to the hydrogen bond presented in water molecules. It gradually increases the solubility of water ions, which affects the physiochemical properties such as TDS, EC, pH, and viscosity [37].

#### 2.3.1. Total Dissolved Solids and Electrical Conductivity

The amounts of TDS and EC present in the NW and MW were measured using a TDS-3 m and HM digital, in which EC was measured in terms of µs/cm, temperature in °C, and TDS in terms of ppm or mg/L. The values of TDS were measured using the direct analysis method. The TDS values were measured as per standard, and the value of the EC may tend to increase gradually with a higher concentration of TDS, which enables the relationship between TDS and EC to be proven [38].

#### 2.3.2. pH

A pH meter was used to measure the hydrogen ion activity in water before and after magnetization using a pH 101 model. As per the procedure elaborated in [38], the pH value was measured for all the samples.

#### 2.3.3. Viscosity

Viscosity is a resistance of the fluid to flow. The higher the amount of internal friction, the higher the viscosity of the fluid, resulting in a large amount of internal friction. The viscosity and the flow rate are interrelated. If the viscosity is high, the flow rate is low and vice versa. Once the water sample was poured into a setup, a stopwatch was switched on until the water reached a 30 cm height and flowed through the capillary tube. If the fluid was more viscous, the flow time would be higher and the flow rate would decrease. The viscosity was measured for the NW and MW at different time intervals using Poiseuille’s capillary tube.

### 2.4. Optical Properties

The optical properties define the absorption and scattering nature of the water medium. If there is any light, it must be absorbed or scattered. The NW and MW light spectrums and their features were observed using advanced instruments such as UV and XRD. The movement of molecules and atoms of the samples were captured based on the spectra, and the corresponding information was filed.

#### 2.4.1. X-ray Diffraction (XRD) Analysis

XRD is a standard method used to identify the structure of atoms or molecules by using beam diffraction. This process was executed using a power X-ray diffractometer made by Bruker (Bremen, Germany), with a tube voltage of 2.2 kW, a Cu-anode ceramic tube with a 0.02-degree scan length. A working volume of 25 mL sample was separated from a 100 mL beaker, which was maintained at 25 °C, and the magnetized sample had 9000 Gauss that was subjected to injection in an instrument for evaluation as per the procedure given in [39].

#### 2.4.2. UV-Visible Spectroscopy

The UV technique was used to measure the absorption properties of NW and MW using a computerized spectrometer model Rigol Ultra 3660. The UV rays were allowed to pass with a polarization consisting of a 0.5 nm spectrum resolution equipped with a 360 to 1090 nm range. The transmittance was 0.001%, and it was applied to the sample in the name of P and S by varying perpendicular polarized light. These terms remain the same in XRD for sampling a 25 mL sample with a 9000 Gauss magnetic field at 25 °C [40,41].

### 2.5. Properties of Concrete

#### 2.5.1. Materials and Mix Proportioning

Ordinary Portland Cement (OPC) of Grade 53 was used in the experiments, the testing of cement was conducted as per IS 8112:2013 [42], the same was used for casting cubes as per IS 2386: 1963 [43], and cement was mixed with fine aggregate and coarse aggregate of 20 mm size to make concrete. The mix ratio for M20-grade concrete was achieved as per IS 10262: 2009 [36], leading to a ratio of 1:1.82:3.10 with a w/c of 0.5.

#### 2.5.2. Fresh and Hardened Properties of Concrete

The standard slump cone test was conducted as per IS 1199: 1959 [44], which measures the amount of water required to achieve the proper consistency of the concrete mixture to achieve better workability without bleeding and segregation. The compressive strength test was employed on hardened concrete cubes of size 150 × 150 × 150 mm mixed with NW and MW. The concrete cubes were tested after curing in water for 7, 14, 21, and 28 days using a compression testing machine according to IS 516:1995 [45] to evaluate the strength attainment of concrete with respect to time [46,47].

### 2.6. TGA

Thermogravimetric analysis was used to investigate the development of hydration products such as CSH, CH, and CC in concrete specimens prepared using magnetic and normal water using a TG/DTA analyzer (TG-DTA-TWIN; TGA 4000). The 28-days-hardened samples were crushed into powder, passing through a 75 µm sieve. Normal and magnetic water concrete specimens were heated up to 900 °C at a heating rate of 20 °C/min under a pure nitrogen atmosphere. The hydrated cement lost weight from 0 to 200 °C bandwidth, representing water loss from the calcium silicate hydrate (C-S-H) layer. Calcium hydroxide (CH) decomposition declined as weight losses between the temperatures of 450 °C and 500 °C [48]. In the band of temperature range between 27 °C to 600 °C, the amount of water decomposed can be obtained from the weight loss of CH [49]. The amount of CH (%) in the specimens was calculated directly from the TG curve using the following equation (Equation (1)).

(1) $$\mathrm{CH}=WL_{\mathrm{CH}}\left(\frac{MW_{\mathrm{CH}}}{MW_{H}}\right)\;$$

where:

*WL*_CH_—Weight loss of water from CH;

*MW*_CH_—Molecular weight of CH (74.01 g mol^−1^);

*MW*_H_—Molecular weight of H_2_O (18 g mol^−1^).

### 2.7. FT-*IR* Analysis

The number of hydroxide (OH) groups present in the cement particles was characterized through FT-IR spectrophotometer analysis. The FT-IR results were examined for NWC and MWC using FT-IR spectrometers with a resolution of 0.5 cm^−1^, PerkinElmer, Singapore. The concrete powder samples passing through the 75 µm sieve were collected and evaluated under ATR mode, with wavelengths ranging from 8200 to 350 cm^−1^. This characterization technique has greater advantages than the traditional one, including less interpretation and greater precision in the quantity of inspecting the materials.

### 2.8. Scanning Electron Microscope (SEM) Analysis on Concrete Powder Samples

SEM analysis was conducted with the support of a high-energy beam of the electron. They generally create subelectrons and spread electrons and diffractions when subjected to various samples. Based on the property of the sample, the electron beam penetrates deeper, which can be deepened further to obtain a clear vision. Further, the voltage increases, and when the electron beam penetrates deeper using detectors, the images can be captured onscreen. Concrete cubes prepared with NW and MW were scanned with Quanta FEG 250 electron microscopy, the results compared the difference between NWC and MWC, and an addition assured the strength improvement of concrete mixed with MW [50]. Then, the concrete samples were converted into fine micron size by using a ball milling machine.

## 3. Results and Discussion

### 3.1. Physiochemical Properties

The magnetic field senses the proton, which flips the water molecules through MW treatment. As a result, the physiochemical properties of MW concrete change [41].

#### 3.1.1. Total Dissolved Solids (TDS) and Electrical Conductivity (EC) of Water

**Figure 3:**
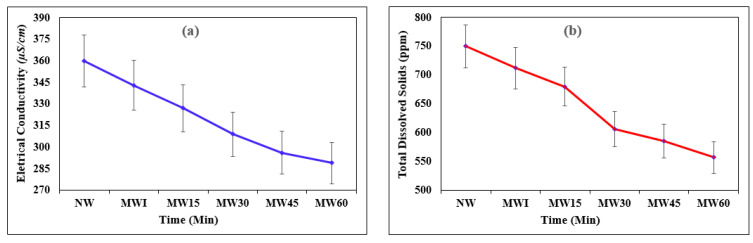
(**a**) Variation in EC and (**b**) TDS of NW and MW with exposure time.

The magnetic field intensity affects the EC of experimental water with exposure. Figure 3 compares the EC and TDS of NW and MW with different magnetic exposures (MWI, MW15, MW30, MW45, and MW60, respectively). From the figure, it can be easily noted that the TDS and EC values were less for the MW than the NW. The amount of TDS present in water decreased when exposed to the magnetic field, and correspondingly, the EC also decreased [38]. Due to the magnetic effect, the values of the EC and TDS decreased up to 19.7% and 25.7%, respectively, when compared to the NW. It can also be understood from the figure that the TDS and EC values reduced tremendously with the magnetic field exposure. Here, the hydration of cement is closely related to the degree of EC of mortar. The EC of mortar depends on mixing water as the water contact is the main reason for dissociating alkali salts in the cement and the calcium hydrate, making them electrically charged ions [51]. Hence, it is possible to correlate cement’s hydration with water’s electrical conductivity.

#### 3.1.2. pH

**Figure 4:**
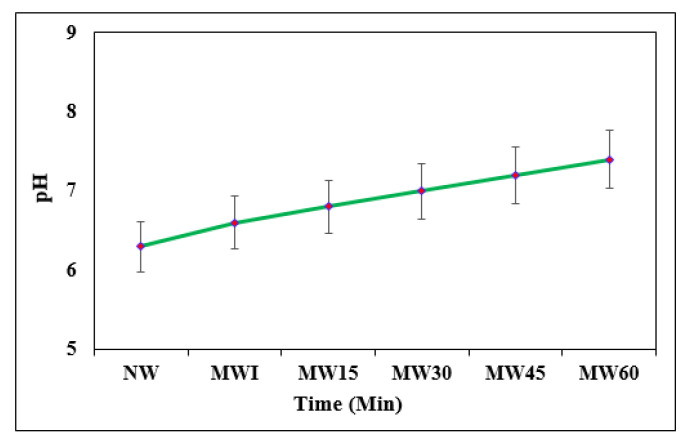
pH variation of NW and MW with exposure time.

The variation in pH impacts the alkalinity value before and after magnetization. Figure 4 displays the difference in pH for NW and MW with different exposure periods varying from MWI to MW60 at 15 min intervals. It was found that the pH of NW was 6.3 and the pH of MW was 7.4 for MW60 exposure. The ions present in the water were responsible for the change in the pH value. In this experiment, the pH value increased with respect to exposure, which indicates that the ${\mathrm{OH}}^{-}$ ions were logically responsible [52]. After applying the magnetic field of 9000 Gauss, there was a formation of calcium carbonate along with other alkalis using hydroxide ions. This naturally increases the pH, which reduces the acidity [53]. With the increase in exposure, there was a significant change in the raising rate of pH. The highest compressive strength was recorded in the alkaline environment, which has a higher surface hardness, and less porosity and hydrated structure compared to the neutral condition [54]. The positive effect on workability and compressive strength of cement can be achieved when the pH is increased to 13 [55]. The increase in the pH value of the water with the increase in the magnetic exposure duration than the normal water is mainly owed to the constant ion product of water becoming affected by the magnetic field, thereby affecting the detachment of the aqueous solution, resulting in enhanced pH values [56].

#### 3.1.3. Viscosity of Water

**Figure 5:**
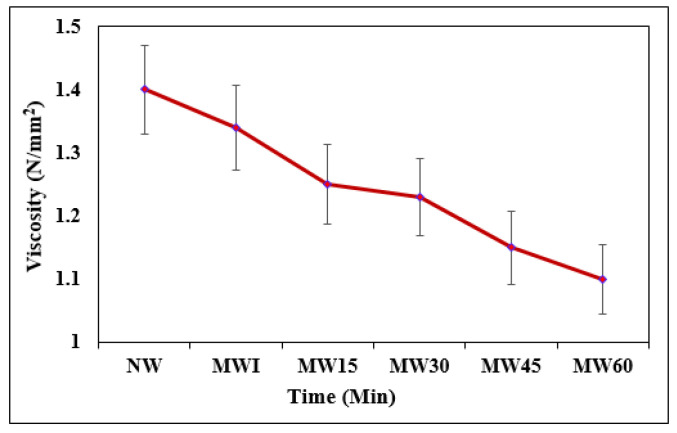
Variation in viscosity of NW and MW with exposure time.

The internal property of the MW enhances the flow rate at different exposures. It can be noticed from Figure 5 that the flow of water significantly increased with exposure to the MW and increased with the exposure period. The fluid accelerated through the 0.9 Tesla magnetic field and increased the flow rate with respect to the increase in exposure. The flow rate of water depends on the size and shape of molecules. Thus, the magnetic field induced on water reduces the amount of internal friction among the molecules and breaks into smaller ones, thereby accelerating the flow rate [34]. An increase in the flow rate of water in the cement paste mixture reduces the permeability and enhances the stiffness of the fresh cement paste mixture [57].

### 3.2. Optical Properties

The magnetic field exposure time for NW was optimized using the TDS, EC, pH, and viscosity measurements. From the outcome of the results, water magnetized for 60 min exposure (MW60) was found to be optimum for the preparation of concrete mixes; hence, the properties were analyzed from the concrete prepared with MW60 water and compared with the properties of concrete prepared with NW.

#### 3.2.1. X-ray Diffraction

**Figure 6:**
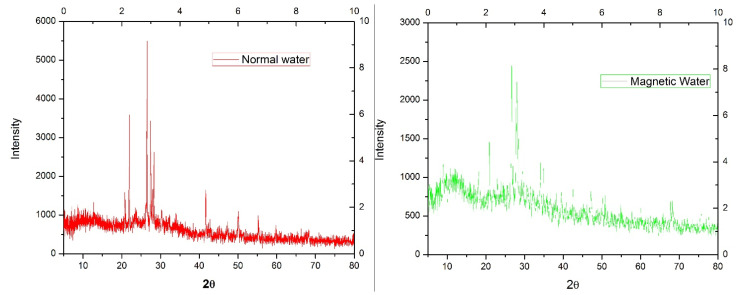
X-ray diffraction spectrum of normal water and magnetic water.

Figure 6 shows that the maximum intensity peaks of XRD for NW and MW lay between the 20 and 30 2θ diffraction angle. By utilizing the magnetic field in water, the diffraction intensity ranges of NW shifted from 2500 to 5565 cps and this drastic transformation may be due to the polarization effect, where the switching of electrons from the atom’s internal structure differs from the normal stage [58].

#### 3.2.2. UV-Visible Absorbance

**Figure 7:**
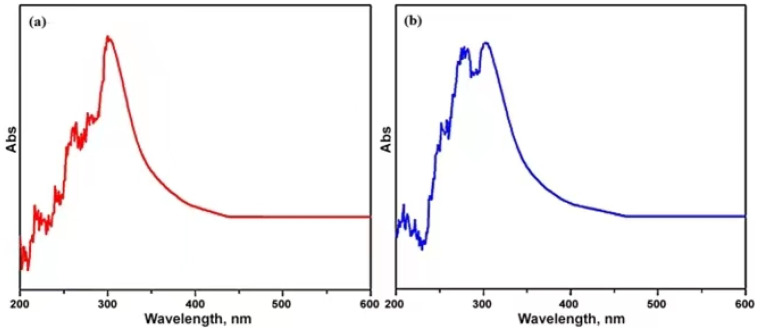
UV-visible absorbance of (**a**) normal water and (**b**) magnetic water.

Figure 7 shows the UV absorption peak intensity in the water medium before and after magnetization. It was observed that the maximum UV absorbance of the magnetic field applied in water ranged between 250 and 300 nm, which indicates that the externally applied magnetic field could only increase the strength of the absorption peak and did not change the position of the peak. Due to the polarization effect, the changes in the structure of the molecules make way for the UV absorbance in a better way for the MW [40].

### 3.3. Fresh and Hardened Properties of Concrete

#### 3.3.1. Effect of Time of Exposure to Magnetic Field on the Slump

**Figure 8:**
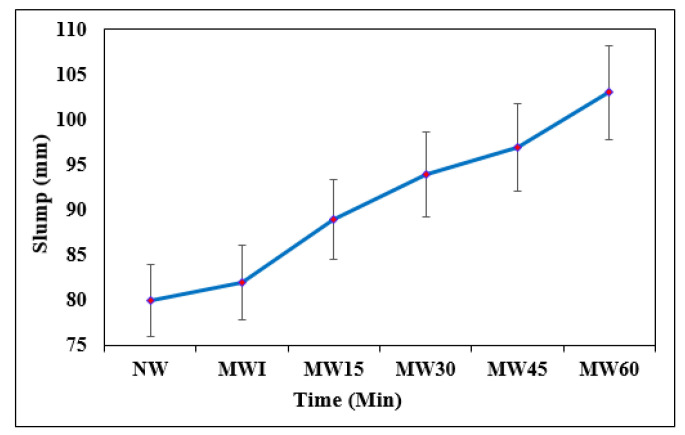
Slump value of concrete with NW and MW at different exposure times.

The slump cone tests are simple and common tests that determine the workability of concrete, and it mainly depends on the water–cement ratio. As per IS 456-2000, a 75 to 100 mm slump is recommended for medium workability, especially for heavily reinforced sections. Figure 8 shows the slump value of the concrete mixes prepared using NW and MW at various exposures. An average of three test data points for each mix were taken for determining the slump value of the mixes. The slump value of the NW mix was found to be 82 mm, whereas for MW60, the slump value was measured to be 103 mm and found to increase with the magnetic field exposure. It was observed that the workability of the developed mixes increased with the exposure period of magnetization. In addition, to check the possibility of demagnetization under vibration, a compaction factor test was carried out, and it was observed that there was no sign of demagnetization of water under vibration, as observed with the insignificant variation in the workability properties of the mixes. The increase in the slump is due to water at the nano-state, which exists in clusters, and this cluster’s size depends upon the dominating force of the water molecules. When water is exposed to a magnetic field, the cluster of molecules is broken by decreasing the bond angle among hydrogen atoms from 104.5° to 103°C due to the macroscopic properties [59]. In addition, during the hydration of cement, the MW percolates through the mid part of the cement composition, which improves the strength of concrete [21].

When NW is used in the preparation of a concrete mix, the hydration process takes place. The products of hydration tend to deposit on the front portion of the cement particles and prevent further hydration in the mix. When a concrete mix is prepared with magnetized water, it allows the passage of well water into the cement particles and enhances the hydration mechanism [60,61,62]. Due to this improved hydration, the properties of the concrete mix are better developed. It has been found that a high degree of hydration of cement could be realized through the application of MW in the concrete mix and that the strength of the concrete could be increased using MW. The increase in the workability with the increase in the magnetic field exposure may also be attributed to the increase in the Lorentz force [63].

#### 3.3.2. Effect on Compressive Strength

**Figure 9:**
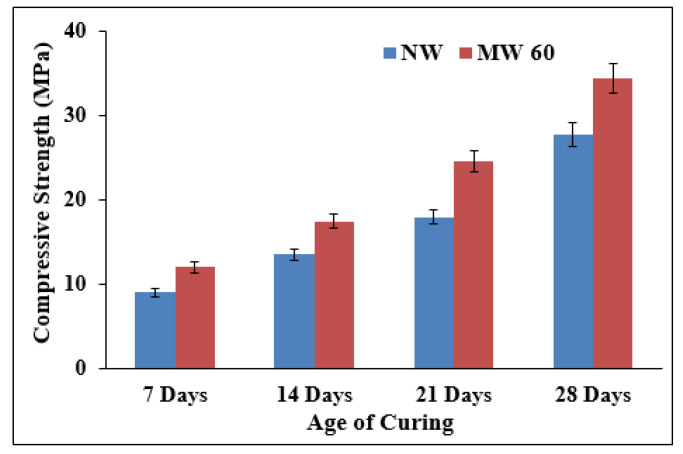
Compressive strength of NWC and MWC.

The concrete cubes were casted with the NW and MW60 and tested for their compressive strength performance. Figure 9 displays the effect of the NW and MW60 on the compressive strength of concrete mixes at 7, 14, 21, and 28 days of curing age. An average of three cube specimens for each mix were taken for determining the compressive strength of the tested mixes. It can be observed from Figure 9 that the mixing of the MW at 28 days improved the compressive strength by 24.1% more than that of the NWC. The target, i.e., the strength of M20-grade concrete, was achieved in 21 days, and hence, the MWC saved the cement content and reduced the curing period. The enhanced strength of the MWC over the NWC may be attributed to a more homogeneous mixture with the MW, which results in the complete hydration of the cement particles. Moreover, it reduces the capillary pores and the discontinuity in packing [64]. The theme behind the MFTW is that the chemical composition (${\mathrm{CaCO}}_{3}$) of scaling is reduced and it produces a greater quantity of smaller water clusters [22,65]. Due to magnetization, water molecules penetrate more easily into the cement particles to stimulate the hydration process of the concrete mix. Subsequently, the mechanical properties of the concrete mix improves. It has been observed that the magnetization effect on the normal tap water can remain for hours or days after magnetization. Hence, magnetic water improves the cement hydration process from the beginning and provides an early strength gain at 3 days. The rate of increase in the strength was found to be more for concrete prepared with MW at later ages than the concrete mixes prepared with NW. This might be due to the distribution of water molecules by the MW, thereby increasing the hydration properties of cement, resulting in improved compressive strength at later ages than at early ages [66]. It was observed that the improved compressive strength of concrete mixes prepared with the magnetic water may be attributed to the larger specific area of magnetized water than the normal tap water [63]. In addition, the magnetized water splits up the heavier water mass into smaller water masses or individual water molecules, allowing the cement particles to fully react with the water. In addition, the hydrogen bonds in the water molecules contribute to the synthesis of hydration products and form a dense C-S-H gel, resulting in enhanced compressive strength. This was also observed by Ghorbani et al. [28] in their SEM micrographs, where they observed additional crystals formation for the mixes prepared with MW than NW.

### 3.4. Thermogravimetric Analysis (TGA)

**Figure 10:**
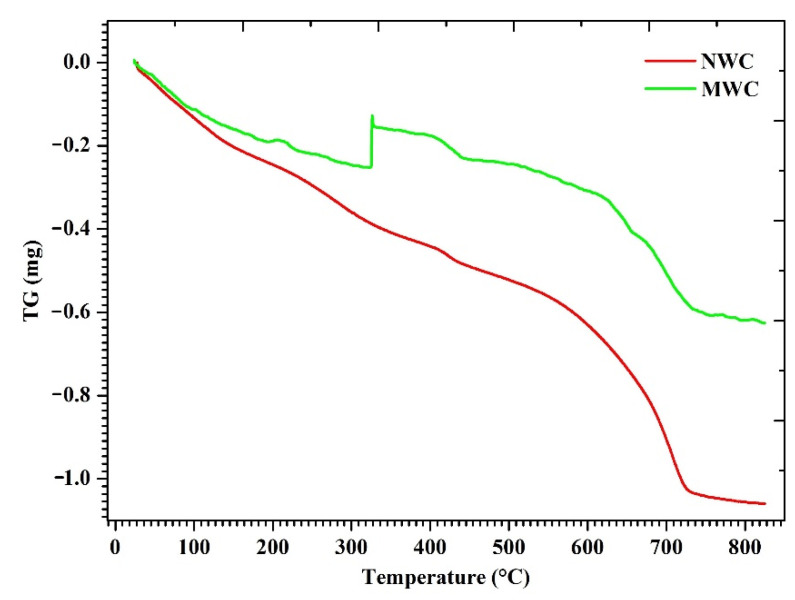
TGA curves for NWC and MWC.

Thermogravimetric analysis was carried out for concrete-powdered samples cast with NW and MW60 after 28 days of curing. From Figure 10, it was inferred that the weight loss observed between the range 110 °C and 300 °C highlighted the dehydration of water molecules allied with C-S-H [67,68]. The weight loss of water molecules associated with calcium hydroxide (CH) ranged between 400 °C and 500 °C [67] for both NWC and MWC. Thermal degradation occurred between the temperature ranges of 600 and 800 °C relating to the de-carbonation of calcium carbonate (CaCO_3_). During the hydration process, CH continuously formed at different ages. It was also observed that MWC showed good resistance with increased exposure than NWC. The weight loss of CH in this bandwidth for NWC was estimated to be 4.31%, whereas concrete specimens incorporated with MW60 resulted in 2.56% loss after 28 days. Due to the magnetic force, water clusters inside the molecules break apart, which accelerates the water to penetrate the cement particles’ core region more easily. The hydration reaction completes, which directly enhances the strength properties of concrete [21]. Therefore, incorporating MW into the concrete mix will improve the cementitious materials’ strength and durability properties.

### 3.5. Fourier Transform Infrared Spectrophotometer (FT-*IR*)

**Figure 11:**
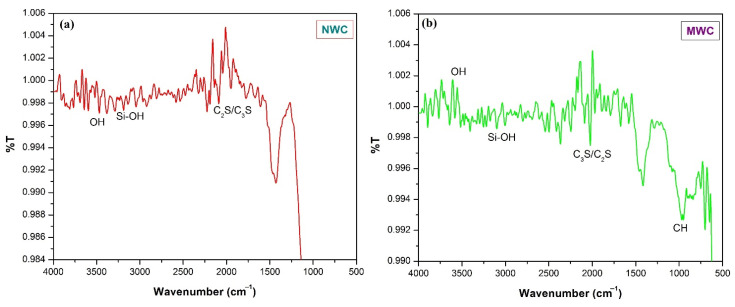
FTIR spectrum of concrete specimens prepared with (**a**) NWC and (**b**) MWC.

FT-IR spectrum analysis was carried out for NW and MW60 concrete mixes after a 28-days curing period, and the results are shown in Figure 11. A band around 3223 cm^−1^ was observed with NW and MW60 and deformation modes of the Si-OH layer in C_3_S and C_2_S [69]. An intense peak around 970–980 cm^−1^ was observed with MWC, which corresponds to a very precise percentage of cement [70]. A peak related to H_2_O appeared above 3000 cm^−1^ (3595 cm^−1^ for MW60 and 3563 cm^−1^ for NW), showing the elongation vibration mode of the OH layer.

### 3.6. SEM Analysis

**Figure 12:**
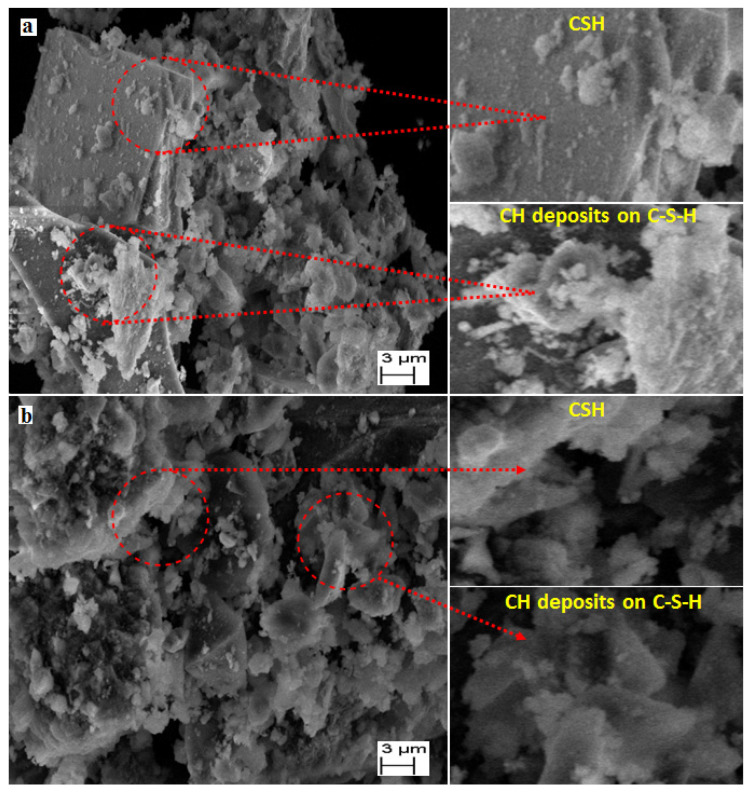
SEM micrograph of calcium hydroxide (Ca(OH)_2_) crystals in cement paste prepared with (**a**) NWC and (**b**) MWC.

Figure 12a,b show the SEM image of the powder samples extracted from concrete mixed with the NW and MW60, respectively, after 28 days of curing. These figures show the availability of calcium hydroxide (CH) crystals in cement paste that were made with both NW and MW60. Samples prepared with the NW (Figure 12a) showed larger CH crystal particles. This is due to the nature of packing in the transition zone followed by the reaction of cement, which forms cluster water molecules. Figure 12b shows the hydrated paste of the CH crystal that was ideal and small. The reaction of the small MW molecules with cement breaks apart into single molecules or smaller ones. Therefore, the activity of water improves. At the same time, the process of hydration benefits the MFTW in a better way that directly enhances the efficiency and strength of concrete [21]. Similar observations were made by Ghorbani et al. [28] with their SEM micrographs, wherein an additional and a compacted crystals formation for the mixes prepared with MW than NW was observed.

**Figure 13:**
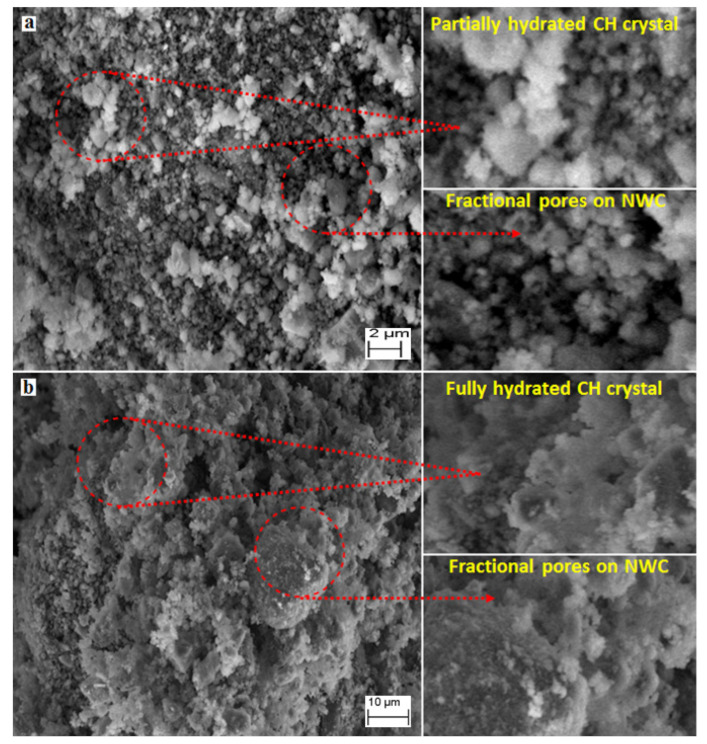
SEM micrograph of fractional voids in cement paste prepared with (**a**) NWC and (**b**) MWC.

Figure 13a, b show the SEM image of the specimens in which the presence of CH crystal and fractional voids were visible, whereas larger voids and a lack of CH crystal had been observed in NWC. However, the MWC showed a higher dispersion of CH crystals and minimal fractional voids that made the structure stronger and capable of resisting cracks [71].

### 3.7. Mechanism

**Figure 14:**
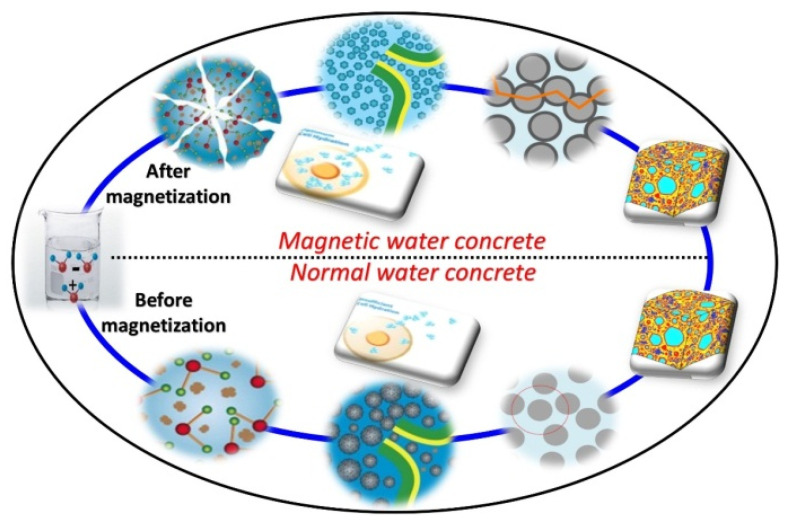
Mechanism of NWC and MWC.

At the nanoscale, water occurs as larger clusters, in which each cluster has 13–15 water molecules. The physiochemical property of the MW becomes altered due to the Lorentz force. In this case, the molecules connected with the hydrogen bond become disconnected, reflecting the orbital motion subject to electrons covering the nucleus of water molecules. Then, $O_{2}^{-}$ and $H^{+}$ are naturally ejected from the bond, which will be the main reason for the enhanced activation of the MW in an isolated performance. The applied magnetic field only affects the physical structure of the water molecules by altering their shape, but its minerals continue to remain constant. As there is a reduction in the size of water molecules, the water layer surrounding the cement becomes thin compared to the NW molecules [72], resulting in reduced water demand for preparing concrete. At the same time, the MW molecules have the potential to penetrate into the cement grains, resulting in improved concrete strength, as shown in Figure 14.

## 4. Conclusions

In this paper, the water quality standards were evaluated using physiochemical properties, namely electrical conductivity, viscosity, pH, and TDS, with the magnetic water at different exposures (MW60, MW45, MW30, MW15, and MWI) and compared with those of the normal tap water (NW). The experimental results showed that the magnetic field improved the physiochemical properties of water with an increase in the exposure time. It was observed that the absorption of MW crystals showed a higher integrity of molecules inside the crystal. It was also shown that the TDS and EC values decreased by 19.7% and 25.7%, respectively, after applying the magnetic field of intensity 9000 gauss. As the viscosity of the MW was lower, it tended to increase the flow rate, and, hence, the workability of concrete improved. The increase in the slump value of concrete with the MWC helped in fixing the cement-to-water ratio, thus reducing the cement content in the concrete. It can be concluded from this research work that the compressive strength of the MWC increased up to 24.1% when compared to the NWC, and the 28-days strength of the NWC was achieved at 21 days itself with the MW. In addition, experiments proved that when the properties of the MW were enhanced, there was an enhancement in cement hydration and workability-related properties. The hydration products of the NW and MW60 concrete mix were characterized through FTIR analysis, and it can be concluded from the TGA results that mixing magnetic water for concreting showed a potential reduction in CH amount and improved the thermal resistance. Microstructure images showed that the concrete mixed with MW provided a fully hydrated CH crystal, and the structure was packed densely. Finally, it was observed that the utilization of the MW for mixing concrete improved the physiochemical properties of fresh and hardened concrete with the minimum usage of water and curing period. Due to water magnetization, the quality of water in the concrete industry improved, which directly enhanced the quality and life span of structures. The need of magnetic water concreting is urgent and there is important demand to construct sustainable building structures with a reduced usage of potable water, thereby increasing sustainability in the construction industry.

## Data Availability

Not applicable.
